# Production of *in vitro *amplified DNA pseudolibraries and high-throughput cDNA target amplification

**DOI:** 10.1186/1472-6750-7-31

**Published:** 2007-06-12

**Authors:** Daniel Frey, Christian Kambach, Michel O Steinmetz, Rolf Jaussi

**Affiliations:** 1 Biomolecular Research, Paul Scherrer Institut, 5232 Villigen, Switzerland

## Abstract

**Background:**

Many structural biology- and high-throughput laboratories experience the acquisition of multiple cDNAs from different sources as a rather time- and resource-consuming procedure. The techniques presented here solve these problems.

**Results:**

An advanced target cDNA amplification procedure employing RNA- or cDNA-derived pseudolibraries circumvents the usual DNA transfection during library establishment. A small sample of reverse transcribed ss- or ds-cDNA or DNA from a pre-existing library is multiplied by in vitro rolling circle ramification amplification. The resulting cDNA pseudolibrary serves as a template for numerous highly efficient PCR amplifications and permits production and analysis of target cDNAs on an automated liquid handling workstation.

**Conclusion:**

The overall efficiency of the simple protocol collection approaches 100% for targets from libraries with low complexity such as Drosophila and yields >80% of amplicons up to 3 kb size in the case of human cDNA.

## Background

The acquisition of a series of full-length cDNAs can be time-consuming and expensive. In a first step, a cDNA clone covering only part of a specific sequence is usually obtained by buying e.g. a clone with an expressed sequence tag (EST). This DNA is then expanded by 5'- and 3' rapid amplification of cDNA ends (RACE) (6). However, the direct acquisition of full-length cDNAs is a difficult task. In our experience, relatively frequently EST clones from academic sources do not contain the target sequence, carry point mutations or represent incomplete splice versions. If clones are bought from a verified source, they are very expensive.

The techniques described in this publication permit the efficient direct amplification and cloning of full-length copies of most desired cDNAs starting from a variety of sequence templates. Moreover, the amplification of target cDNAs in a high-throughput format requires a large amount of template cDNA mixture that is usually purchased in form of an expensive commercial library. Alternatively, an mRNA sample is reverse transcribed to cDNA which then serves as a template for PCR amplification of the targets. In both cases the template cDNA is scarce unless it is cloned in a vector and amplified in *E. coli *cells. This procedure is inevitably accompanied by under-representation or loss of certain cDNA sequences due to unequal transfection efficiency or stability of the different plasmids.

By use of the isothermal rolling circle amplification (RCA) method, the techniques described here can generate ample amounts of template cDNA from any kind of source mRNA/cDNA without noticeable loss of sequence representation. A pseudolibrary, as described here, is generated entirely *in vitro *by a minimized number of experimental steps and omits transfection and amplification of the DNA inside bacterial cell clones. Furthermore, the synthesis of a second cDNA strand ahead of amplification is avoided. The first strand cDNA is synthesized by reverse transcription of mRNA. The RNA strand is removed by denaturation and cleavage with RNAse I. The remaining cDNA is treated with a specific ligase that produces closed circles from linear single-stranded DNA. The circularized ss-cDNA is subjected to RCA using Φ29 DNA polymerase and chemically modified random primers which boost amplification. The direct processing of the first strand cDNA enhances the cloning of "difficult" target sequences. The subsequent RCA generates ample DNA to be used as template for high-throughput target amplification by PCR. Finally, an in-house developed and exceptionally efficient cDNA amplification protocol using PCR with Phusion DNA polymerase leads to capture of the targets upon the first attempt with a success rate of 80% or more.

In our approach, the combination of jointly optimized steps fuses several recently available techniques to a powerful cDNA-amplification and -cloning ensemble useful in high-throughput settings like those heading for protein production for structural biology or other demanding applications.

## Results

### Plasmid cDNA library quality and success of amplifications

**Figure 1:**
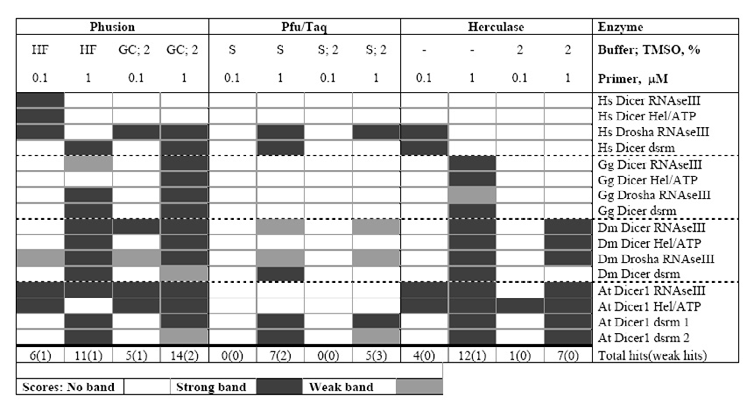
**Result summary of gel electrophoreses with PCR products**. Pictures of the gels were taken after staining with ethidium bromide. They were visually inspected and bands with suitable DNA size were scored (cf. fig. 2). Species identifiers: Hs, Homo sapiens; Gg, Gallus gallus (chicken); Dm, Drsosophila melanogaster; At, Arabidopsis thaliana. Domain abbreviations: Hel, helicase; ATP, ATPase, dsrm, double-stranded RNA-binding motiv. Designations: Species identifier, protein name, short designation of target domain(s).

**Figure 2:**
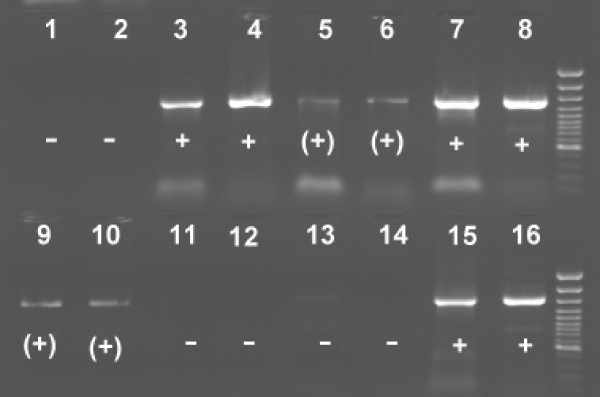
**Agarose gel of PCR products from the Drosophila pseudolibrary**. 5 μl samples of the PCR reactions were run on a 1.2% Agarose gel in 1 × TBE for 30 min. The DNA-bands were scored as indicated. Lanes: 1–8 Dicer 1 RNAse III; 9–16 Dicer 2 RNAse III; 1,13 Herculase 100 nM primers; 2,9,14 Phusion HF buffer, 100 nM primers; 3,15 Phusion HF buffer, 1 μM primers; 4,16 Phusion GC-TMSO buffer, 1 μM primers; 5 Pfu/Taq 1 μM primers; 6 Pfu/Taq-TMSO 1 μM primers; 7 Herculase 1 μM primers; 8 Herculase-TMSO 1 μM; 10 Phusion GC-TMSO buffer, 100 nM primers; 11 Pfu/Taq 100 nM primers; 12 Pfu/Taq-TMSO 100 nM primers. HF- and GC-buffers are intended to favor high-fidelity PCR of templates with low or high GC-content, respectively. Markers: 100 bp DNA ladder Plus (Fermentas), top band 3000 bp, strong band 500 bp.

Because of the high copy fidelity of Phi29 DNA polymerase, the success of target cDNA amplification is primarily determined by the quality of the source mRNA or cDNA. The MegaMan library (Stratagene) proved to be an excellent source for human cDNAs. The RCA product permitted us to amplify over 80% of all desired human cDNA targets at the initial attempt (fig. 1, fig. 2 and additional data, not shown). The primer design was carried out with Vector NTI (Invitrogen) choosing Tm values around 55°C (probe 250 pM, salt 50 mM) or with the primer selection tool of the Joint Center for Structural Genomics of the NIH. Both primer designs yielded similar results and are not distinguished in the table. Corresponding cloning experiments applying standard conditions as suggested by the suppliers with Vent polymerase (NewEngland Biolabs) or a mixture of Pfu polymerase and Taq polymerase (2.5 U Pfu + 1 U Taq per 50 μl PCR reaction) in Pfu buffer (Stratagene) gave significantly fewer positive PCR reactions (50–60%). Variation of the conditions such as adding DMSO, TMSO or changing the Mg-Ion concentration did not improve the percentage of positive reactions.

The other cDNA pseudolibraries displayed a quality similar to that of the amplified MegaMan library, we could obtain 80–100% of the desired targets at first attempt. Only a single target class proved to be notoriously difficult; we could not obtain amplification of any full length cDNA of the plexin family from the chicken library. These cDNAs span 6–10 kb and thus are unusually long. However, primers for shorter plexin fragments led to amplification of the expected fragments. Somewhat shorter targets like semaphorin domains of about 3–4 kb length were amplified with a success rate of about 50%. Not surprisingly, the amplification of very long cDNAs required amplicon splitting or further optimization of the PCR conditions.

### Systematic study of a series of amplifications by robotics

Two genetically engineered DNA polymerases containing accessory DNA binding domains (Phusion DNA polymerase and Herculase II Fusion polymerase) were compared with a mixture of classically used DNA polymerases (Pfu plus Taq). The amplification conditions were kept as similar as possible. All PCR reactions were run at the same primer concentrations and in presence or absence of 2% TMSO. The thermocycler program was also the same for all amplifications: 30 s at 95°C, 1 min at 65°C with 0.5°C decrease per cycle, 30 s (fusion polymerases) or 1 min (Pfu/Taq) per kb of the target at 72°C for 20 cycles; then 20 cycles at 95°C for 30 s, 30 s at 52°C, with the same extension time.

Each primer contained an attB1 or attB2 site, respectively, to permit downstream cloning by the Gateway technology (for details cf. InVitrogen Gateway manual). The results upon agarose gel electrophoresis (for an example, see fig. 2) were scored by inspection of the gels for presence or absence of a band with the expected size (data summary in fig. 1). Strong and weak bands were discriminated visually. A strong band indicated that the cloning of the fragment by Gateway technology was highly probable; over 90% of the entry clones verified by sequencing showed the expected sequence.

Recapitulating we can state that the fusion DNA polymerases perform much better than the mixture of the conventional enzymes Pfu/Taq (Tab. 1): Out of 64 tested cases Phusion polymerase was positive in 36; Herculase in 24 and Pfu/Taq in 12. Only Phusion polymerase could amplify all of the 16 tested targets, Herculase failed in 2 cases and Pfu/Taq in 9. The results described here are confirmed by many more target amplifications that were handled manually in our laboratory. We experience that over 80% of the targets will be amplified at first attempt when using the four Phusion polymerase conditions. In organisms of lower genetic complexity like Drosophila we obtained all targets at first go. At this time point we have set up amplifications for totally 71 targets in four species and have obtained 65 positive PCRs.

## Discussion

Rolling circle amplification is an established technique for amplification of small samples of template DNA mixtures [1-4]. The technique is well established and known to yield amplified DNA with high fidelity and without loss of sequence representation [1,2,5,6]. It has been mainly used for amplification of genomic DNAs or sequencing templates, but was so far not systematically applied for expansion of mixtures of ss-cDNA to be followed by PCR amplification and cloning of target cDNAs. This paper describes several variations of this kind of application: I) PCR template generation via poly(A)^+^mRNA or total RNA, reverse transcription and second strand synthesis yielding ds-cDNA followed by RCA and high-efficiency PCR; II) PCR template generation via poly(A)^+^mRNA or total RNA, reverse transcription to ss-cDNA, circularization of ss-cDNA by direct ligation, RCA and high-efficiency PCR. High-throughput cloning of cDNAs requires relatively high template quantities because rare transcripts should be amplifiable.

Upon production of genomic libraries employing rolling circle amplification, the rearrangement of DNA has been observed [7]. The majority of these chimeras are inverted sequences with an intervening deletion [8]. Such artifacts will probably not occur during the amplification of cDNA as described in this paper, because the chimeras are formed during the cloning steps inside *E. coli *cells [7] while our method comprises only *in vitro *amplification steps.

The procedures had to be optimized to achieve a good overall success; especially the final DNA purification steps proved to be critical in this respect. The PCR enzyme(s) and the amplification protocols also needed to be adjusted to the template in order to permit highly reliable cloning of target cDNAs from complex mixtures like mammalian cDNA pseudolibraries. We thus strongly recommend using the protocols described here as an entity.

## Conclusion

The presented set of methods means saving time and money. The practical production of a specific cDNA in the laboratory is less time consuming than the ordering and mailing of cDNA clones from external sources. In our experience, the acquisition of clones from pre-existing libraries can fail in a considerable percentage of cases, and buying verified clones is rather expensive. We thus try to acquire cDNAs by the described methods before we access outside sources. The techniques required for the insertion of the amplified cDNAs into plasmid vectors are well established and for this reason have not been described here. The current methods of choice are the Gateway technology (Invitrogen), the StrataClone topoisomerase-based cloning kit or the InFusion cloning technique (Clontech). Employing a pseudolibrary as template for target amplification with Phusion polymerase is thus established as an extremely useful combination to rapidly amplify multiple target cDNAs at low cost.

## Methods

### General remarks

All general molecular biology techniques were carried out as described in the Molecular Cloning Laboratory Manuals by Sambrook and Russell (Cold Spring Harbor Laboratory Press). DNA- and RNA-concentrations were measured in one-microliter samples using a NanoDrop spectrophotometer (NanoDrop Technologies, Wilmington, Delaware, USA).

### Template libraries and RNAs

The MegaMan library (Stratagene, La Jolla, CA, USA) is a transcriptome library which contains pooled plasmids harboring cDNAs from many different human tissues and cell lines. Chicken total RNA was prepared from frozen E5–E9 stage chicken embryos (supplied by Esther Stöckli, University of Zürich). The RNA was purified with Ambion's MELT total RNA preparation system (Ambion, Austin, TX, USA). The other RNAs were purchased from commercial sources; adult *Drosophila melanogaster *poly(A)^+ ^mRNA was from Clontech, BD Biosciences (Palo Alto, CA, USA) and *Arabidopsis thaliana *total mRNA from BioChain Institute (Hayward, CA, USA).

### Construction of template for PCR amplification from plasmid cDNA library

The MegaMan plasmid cDNA library was amplified by RCA with the bacteriophage Φ29 DNA polymerase [9]. The random octameric oligonucleotide primers were modified with two Nitroindole bases and two phosphorothioate links: 5' Nitroindole-Nitroindole-NNNN-s-N-s-N 3' (N = any base, s = phosphorothioate link; obtained from Microsynth, Balgach, Switzerland) in order to increase template-specific synthesis while keeping the background at a low level [2]. A 180 microliter reaction contained 0.1 mM random primers, 75 ng cDNA library, 1 × Tango buffer (33 mM Tris-acetate pH 7.9 at 37°C, 10 mM Mg-acetate, 66 mM K-acetate, 0.1 mM BSA, Fermentas, Vilnius, Lithuania), 0.4 mM desoxynucleoside triphosphates, 1 × Φ29 polymerase buffer, 2.5 mU ~ 1.3 μg yeast pyrophosphatase (Roche Diagnostics, Basel, Switzerland) and 35 U Φ29 DNA polymerase (New England Biolabs, Ipswich, MA, USA or Fermentas). The mixture was incubated for 24–48 h at 34°C, thereafter EDTA was added to 20 mM. The stopped reaction was subjected to 3 freeze-thaw cycles to shear the large DNA complexes and precipitated with 2.5 volumes of ethanol in the presence of 2 M ammonium acetate pH 5.0 for 15 minutes on ice. The precipitate was spun down for 10 minutes at 4°C at 13'000 × g. The pellet was washed 2 × with 70% ethanol, dried for 5 min at room temperature and dissolved with 500 μl 10 mM Tris-Cl pH 8.0, 1 mM EDTA during 30 min at 60°C. Thereafter, the solution was kept overnight on a turning wheel at room temperature. This procedure yields at least 150 μg DNA, i.e. sufficient template DNA for 1000 standard 50 μl PCR reactions (see below). Other ways of purifying the DNA, e.g. spin column adsorption and elution, resulted in loss of most of the large branched DNA complexes; e.g. the purification of the DNA using QIAquick PCR purification columns (Qiagen) generated tenfold less DNA (cf. [10]).

### Construction of template for PCR amplification from poly(A)^+^- or total RNA via ds cDNA

The first strand cDNA was synthesized using a cDNA synthesis kit (Roche). The cDNA was cleaned by phenol-chloroform extraction and ethanol precipitation in presence of 2 M ammonium acetate pH 5.0. The products obtained from 1 μg of poly(A)^+ ^RNA or from 10 μg of total RNA were dissolved in 25 μl of 10 mM Tris-Cl pH 8.0, 0.1 mM EDTA. The double-stranded cDNA was ligated to concatemers and circles with T4 DNA ligase in presence of 5% polyethylene glycol 4000 according to the specifications of the supplier (Fermentas). The overall yield was 1–2 μg ligated cDNA. Approximately 100 ng of this cDNA were amplified with Φ29 DNA polymerase as described above and yielded template for about 1000 PCR reactions.

### Construction of template for PCR amplification from poly(A)^+^RNA or total RNA via direct circularization of the ss cDNA

The oligodT(15) primer was ordered as a 5' phosphorylated primer (Microsynth) or was treated with T4 polynucleotide kinase and rATP (Fermentas). The phoshorylated primer was annealed to the chicken embryonal total RNA (4–6 μg) and the first strand cDNA synthesized with AccuScript reverse transcriptase (Stratagene) according to the specifications of the supplier. EDTA was added to final 20 mM to the ss cDNA product (about 3–5 μg cDNA in 40 μl), heated to 98°C for 2 min to melt the cDNA-RNA hybrids. The solution was chilled and 15 U RNAse I (from the Roche cDNA synthesis kit) was added. After 30 min at 37°C, 3 U Proteinase K were added and the incubation was continued at 37°C for 30 more minutes. Thereafter, Proteinase K was inactivated by heating at 70°C for 15 minutes. The products were precipitated with ethanol and 2 M ammonium acetate pH 5.0. After the wash with 70% ethanol, the accrued 2.5–3.0 μg DNA was dissolved in 15 μl H_2_O. To this sample, 2 μl of Circle Ligase buffer, 50 μM rATP and 2 μl of CircLigase (Epicentre, 100 U/μl) were added. The ss cDNA was circularized by incubation for 1 h at 60°C and 10 min at 80°C. This circular DNA could be used for ramification amplification with Φ29 DNA polymerase as described above. A sample containing 400 ng of circular ssDNA yielded about 80 μg ds DNA to feed about 1000 standard PCR reactions with template.

### Target amplifications

When the novel Phusion DNA polymerase PCR mixture (Finnzymes) became available, we decided to compare its performance with that of the most successful PCR set up known to us. Its application according to the supplier's specifications (both HF and GC buffer) improved our results from about 50 to 70% positive reactions at first go. Both addition of methyl sulfoxides [11] and changing the primer concentration, are known to largely influence the PCR outcome. When tetramethylene sulfoxide (TMSO, Acros Chemicals) was introduced as an additive with high and low primer concentration (1 μM or 100 nM final concentration, respectively) we achieved the highest level of positive reactions. The most successful combinations required four separate PCR reactions per target: a) high primer, HF buffer, b) low primer, HF buffer c) high primer, GC buffer, 2% TMSO, d) low primer, GC buffer, 2% TMSO. Tests with commercial PCR optimization kits like the FailSafe PCR system (Epicentre, 12 different conditions) did not outperform the a/b/c/d conditions with Phusion polymerase. The cycling parameters are also crucial. The most reliable result was obtained using a step-down protocol: 1 min 98°C; 20 cycles 30 s 98°C, 1 min 60°C to 50°C (decrease of 0.5°C per step), X min 72°C (depends on target length, about 20 s per kb) followed by 20 cycles 30 s 98°C, 30 s 52°C, 1 min 72°C. Finally cool to 10°C. The PCR products are stable at this temperature for a few days. For the classical DNA polymerases (Pfu plus Taq) the extension time was 1 min per kb.

We set up a systematic survey comparing the Phusion polymerase conditions with the well-known Pfu-Taq mixture and one of the most recently available other fusion polymerases, namely Stratagene's Herculase II Fusion polymerase. The three polymerase formulations were used at high or low primer concentrations (1 μM or 100 nM, respectively) as well as in absence or presence of 2% TMSO (the maximally tolerated TMSO concentration with Phusion polymerase is about 6%, data not shown). To ensure highly reproducible pipetting, we ran the experiments on a Tecan Freedom Evo II liquid handling workstation. The master mixes including the standard buffer, enzyme(s), nucleotides and water to 50% of the final volume were mixed manually. The remainder was added by the liquid handling workstation. The PCR was carried out by an MJ Research (BioRad) thermocycler installed on the deck of the machine. The agarose gels were prepared and run manually or were E-gels (InVitrogen) and run by the workstation.

## Authors' contributions

The presented work is clearly a team's effort: Daniel Frey (Tecan workstation), Christian Kambach (structural biology) and Rolf Jaussi (gene technology) contributed each a major part to the experimental work. Michel O. Steinmetz (structural biology) supported us a lot with ideas, detailed technical discussions and by helping us writing the manuscript. He is responsible for the acquisition and supervision of the installation of the Tecan Freedom Evo II workstation. All authors joined many fruitful discussions.
